# Extraction of non-infected redundant pacing and defibrillator leads does not result in better patient outcomes

**DOI:** 10.1007/s12471-023-01770-7

**Published:** 2023-04-03

**Authors:** Frank A. Bracke, Leonard M. Rademakers, Dennis van Veghel

**Affiliations:** Department of Cardiology and Cardiothoracic Surgery, Catharina Hospital, Eindhoven, The Netherlands

**Keywords:** Lead extraction, Pacemaker, Defibrillator, Abandoned, Complications, Indication

## Abstract

The introduction of dedicated tools for pacing and defibrillator lead extraction has resulted in relatively high success and low complication rates. The confidence this elicits has broadened the indications from device infections to non-functional or redundant leads and the latter make up an increasing share of extraction procedures. Proponents of extracting these leads point to the higher complication burden of lead extraction in patients with longstanding abandoned leads when compared one-to-one with extraction when these leads become redundant. However, this does not translate into better patient outcomes on a population level: complications are rare with properly abandoned leads and thus most patients will never be subjected to an extraction procedure and the ensuing complications. Therefore, not extracting redundant leads minimises the risk for the patients and avoids many expensive procedures.

## Introduction

When pacing or defibrillator leads become redundant or defective, one has the choice between lead abandonment or lead extraction. Abandonment can be performed by any device specialist, and the only additional cost is an end cap or a non-absorbable suture to secure the lead in the pocket. In the case of lead extraction, contrastingly, patients must be referred to specialised centres and the procedure is expensive. This is not only due to dedicated and expensive tools, but extraction also has to be performed in (hybrid) operating rooms with a complete cardiac surgery team on standby and with the backup of an intensive care unit in case of complications when performed according to the guidelines [1].

Nonetheless, especially in high-volume centres, almost half the indications for lead extraction in recent publications concern redundant or malfunctioning leads. This practice can only be justified when the higher expenditures are counterbalanced by better patient-relevant outcomes.

## Discussion

The 2017 Heart Rhythm Society (HRS) expert consensus statement on cardiovascular implantable electronic device lead management and extraction (endorsed by the European Heart Rhythm Association) supports both abandonment and removal as useful treatment strategies if a lead becomes clinically unnecessary or non-functional with a Class IIa indication [1]. The consensus document regards extraction of unnecessary hardware beneficial as “a lead will never be easier to extract than it is today”. The document stresses that decisions should be made in an informed discussion between operator and patient. But how should the patients be informed? There are no randomised trials in support for either option, and none of the referenced small, non-randomised studies yield a better outcome with lead extraction. The expert’s motivation is derived from comparisons between procedural outcomes of lead extraction in patients with previously abandoned leads and the outcomes of extraction in patients without abandoned leads [2, 3]. For instance, the Cleveland Clinic reported 3.7% major complications from extraction in patients with abandoned leads compared with 1.4% in their overall population with only active leads [2]. The authors’ conclusion was: “The study highlights the importance of resisting the expedient clinical decision of abandoning leads at the time of CIED system change, revision, and upgrade”. So, from a procedural standpoint, early extraction seems the sensible option, and patients are often advised in that direction.

However, a first caveat is the difference in dwell time: this was twice as long in patients with abandoned leads compared with patients with only active leads, and this greatly influences outcome. A second caveat is that when discussing the options with the patient, the risk of direct extraction of non-functional leads is often downplayed. The Cleveland Clinic, however, also published two reports about extracting non-functional leads at the time of device upgrade or lead revision. The first one reported on 503 procedures with 1.0% major complications, including 2 deaths, the second on 430 procedures for recalled Fidelis or Riata leads with 1.5% major complications and a 0.7% mortality incidence [4, 5]. Likewise, three other high-volume US extraction centres with a combined experience in 891 extraction procedures of Fidelis and Riata leads reported 1.3% major complications and a 0.65% mortality [6]. These results are in spite of a mean or median implant time of less than five years in all three mentioned studies. Also a quarter of the major complications in 2999 extraction procedures in the Cleveland Clinic occurred in procedures with a less than five years combined age of the extracted leads [7]. This illustrates how difficult it is to define a truly low-risk extraction procedure. Although leads implanted less than a year ago can nearly always be removed with simple traction, the odds diminish rapidly with longer dwell times. Therefore, as a rule, we refrain from removing leads if they have been implanted more than two years ago, or—with shorter implant times—if the lead body is not completely mobile inside the vasculature. There is a danger that after two years, leads may effortlessly detach from the myocardium but get trapped in more proximal scar tissue and one still must use extraction tools but in a less favourable situation.

A third caveat is that authors compare lead extraction in patients with longstanding abandoned leads one-to-one with extraction when these leads become redundant. This implies the unsupported assumption that most patients with abandoned leads eventually need extraction procedures. However, the available literature indicates that the incidence of complications in patients with abandoned leads is comparable to patients with active leads given similar dwell times and device complexity, and there is no greater need for lead extraction in this population. The Mayo Clinic retrospectively reviewed patients with abandoned leads from 1977 till 1998 [8]. They reported complications in 24 of 433 patients: infection in 8 (1.8%) and asymptomatic venous occlusion at the time of a new lead placement in 16 patients. Lead extraction was eventually performed in 15 patients: in all patients with infection and in 7 with an obstructed vein to regain venous access. The same institution specifically followed 78 patients with 101 abandoned defibrillator leads [9]. No indication for implantable cardioverter-defibrillator (ICD) related surgery during follow-up was attributed to abandoned leads. The authors concluded that abandoning a non-functional lead is safe and does not pose a clinically significant additional risk of future complications. In the Danish Pacemaker and ICD Registry 5.5% of 740 patients with abandoned defibrillator leads underwent lead extraction for device infection during a follow-up for 4.4 ± 3.1 years [10]. This is comparable with the 2.3 to 5.0% infection rate depending on the type of device in the overall Danish ICD device cohort [11]. Alqarawi et al. reported their follow-up in 213 patients with abandoned Fidelis leads: there were 1.4% pocket infections requiring extraction and no deaths during follow-up [12]. Finally, there is no proof available that extraction of non-functional leads results in fewer future complications, including the need for a possible second lead extraction, or that implanting new leads is safer after extracting non-functional leads.

Therefore, as only a small number of patients with abandoned leads eventually require lead extraction, and even though the complication rate may be higher at that time, the total number of complications will never surpass the total number of extraction-related complications when all patients with non-functional leads are subjected to extraction.

Proponents for extraction may refer to the consensus statement for additional arguments: preservation of access to magnetic resonance imaging (MRI), creation of an access through an occluded vein to allow a new lead to be implanted and safeguarding tricuspid valve function from damage by multiple leads [1]. However, MRI in the presence of abandoned leads is not contraindicated anymore since the 2021 European Society of Cardiology (ESC) guidelines changed the recommendation from Class III to Class IIb [13]. In contrast, residual lead fragments after extraction in about 4% of patients will result in a Class III recommendation for these patients [14].

Regaining vascular access through extraction can be a valid option, but preventing venous occlusion by extracting superfluous leads is an unproven concept as extraction has been shown to damage the veins with possible venous thrombosis as a result [15, 16]. Still, venoplasty is a very safe procedure compared with extraction and is the preferred option any time a guidewire (and balloon) can cross an obstruction.

The arguments for severe tricuspid regurgitation from multiple leads passing the valve are limited and conflicting [17]. In contrast, lead extraction has been associated with significant new or increased tricuspid regurgitation in 12–14% of patients [18, 19].

Finally, one should realise that many complications are not caused by abandoned leads, but by the manner they are abandoned. Lead migration, skin erosion and electrical interference can all be avoided by adequate surgical techniques and properly securing and insulating the leads in the pocket. For example, Kirkfeldt et al. showed that for pacing devices complications during upgrade or lead revision are lower than with primary implants, but the reverse is true for ICD devices [20]. Enlarging existing pockets, relocating the device to a submuscular position if necessary and avoiding unnecessary bulk in the pocket by severing connectors of abandoned leads (instead of using end caps) is more important than extracting non-functional leads to avoid complications.

## Conclusion

There is no evidence that extraction of non-functional or redundant leads improves the patient’s outcome, but it does expose them to unnecessary risks. Although decisions about extraction should be made in a team involving cardiologists, cardiac surgeons, intensive care specialists and infectiologists if applicable, it does not alter the evidence that adequately abandoning leads has a demonstrated lower complication rate without jeopardising the prognosis for the patients. A consequence of forsaking extraction of non-functional leads will be a lower demand for extraction procedures and this may have repercussions for maintaining the experience of all current extraction centres.

In conclusion, there should be the utmost constraint to extract non-functional leads, both from the perspective of patient-relevant outcomes and sensible healthcare expenditure. Device-related infections are and should remain the only important indication for lead extraction.
